# Effect of Grape Polyphenols on Blood Pressure: A Meta-Analysis of Randomized Controlled Trials

**DOI:** 10.1371/journal.pone.0137665

**Published:** 2015-09-16

**Authors:** Shao-Hua Li, Peng Zhao, Hong-Bo Tian, Liang-Hua Chen, Lian-Qun Cui

**Affiliations:** Department of Cardiology, Shandong Provincial Hospital Affiliated to Shandong University, Jinan, China; University of Catanzaro Magna Graecia, ITALY

## Abstract

**Background:**

The effect of grape polyphenols on blood pressure remains unclear, which we aimed to address via a meta-analysis study.

**Methods:**

We conducted study trial searches in PubMed, Embase, and the Cochrane Library databases. Summary estimates of weighted mean differences and 95% confidence intervals were obtained by using fixed-effects models. Subgroup analyses were performed to identify the source of heterogeneity. The protocol details of our meta-analysis have been submitted to the international database of prospectively registered systematic reviews (registration number *CRD42015019196*).

**Results:**

Ten studies were included in the present meta-analysis. Our results showed daily grape polyphenol intake could significantly reduce systolic blood pressure by 1.48 mmHg when compared to control subjects (12 comparisons; -1.48 [-2.79 to -0.16] mmHg; *P* = 0.03). Subgroup analyses indicated larger reduction was identified in the intake of low-dose of grape polyphenols (< 733 mg/day, median level of the included studies) or patients with metabolic syndrome. Contrarily, diastolic blood pressure was not significantly decreased in the grape polyphenols group as compared to controls. No significant heterogeneity or publication bias was detected in the meta-analysis of either systolic or diastolic blood pressure.

**Conclusions:**

Daily grape polyphenol intake can significantly reduce the systolic blood pressure in humans, although the reduction is modest when compared with anti-hypertensive medications. Larger, better designed trials, that specifically include hypertensive subjects, are required to verify our results in the future.

## Introduction

Blood pressure is a pivotal parameter of the cardiovascular system, and hypertension is an important risk factor for cardiovascular diseases [1]. A previous study revealed that a 4-5mmHg reduction in systolic blood pressure and a 2-3mmHg reduction in diastolic blood pressure could significantly reduce cardiovascular risk by 8%-20% [2]. In addition to anti-hypertensive medications, improvement of blood pressure by dietary interventions has been recommended by the American Heart Association [3].

Grape products contain polyphenols, such as anthocyanins, flavanols and flavonols, as well as phenolic acids [4]. Experimental studies have indicated that these polyphenols may increase nitric oxide (NO) bioavailability, improve insulin sensitivity, antioxidant protection, and decrease blood viscosity [5–7]. In vivo animal studies have further demonstrated that grape polyphenols can induce an endothelium-dependent relaxation in rabbits, [8] and reduce arterial blood pressure in hypertensive rats [9]. However, the precise effect of grape polyphenols on blood pressure in humans has not been well clarified. Some studies reported that daily oral grape polyphenol intake could significantly decrease systolic or diastolic blood pressure [10–14]; whereas others showed that blood pressure might be not changed, and even increased, after daily grape polyphenol supplementation [15–19].

Feringa et al. included 5 trials (until 2010) and conducted a meta-analysis investigating the effect of grape polyphenols on blood pressure [20]. However, one important trial published in 2008 [10] was not included in Feringa's meta-analysis, and significant publication bias could be found in the analysis of diastolic blood pressure. Furthermore, many new clinical trials have been published since 2010 [12,14,17,19]. Therefore, a new meta-analysis is necessary to explore the precise effects of grape polyphenols on blood pressure in humans. In the present study, we identified all published, randomized, and controlled trials of grape polyphenols and performed a meta-analysis to evaluate the effect of grape polyphenols on systolic and diastolic blood pressure in human subjects.

## Materials and Methods

The present meta-analysis was conducted according to the Preferred Reporting Items for Systematic reviews and Meta-Analyses (PRISMA) guidelines [21]. The protocol details and the PRISMA checklist have been provided in S1 and S2 Tables. Meanwhile, the protocol details of our present meta-analysis have been submitted to the PROSPERO register and this record has been published on the database at http://www.crd.york.ac.uk/prospero/. Our registration number is *CRD42015019196*.

### Search strategy and selection criteria

According to PRISMA guidelines, we systematically searched PubMed (from 1950 to March, 2015), EMBASE (from 1966 to March, 2015), and the Cochrane Library for published reports by using the query “(grape) OR (polyphenol)” paired with “(blood pressure) OR (hypertension).” In addition, a manual search of references from reports of clinical trials or review articles was performed to identify relevant trials. When applicable, attempts were also made to contact investigators for clarification or additional unpublished data.

Studies were selected for analysis if they met the following criteria: (i) the article was published in English; (ii) study was a randomized and controlled trial in humans; (iii) the subjects in the trial were exposed to the intervention for a minimum of 2 weeks; (iv) means of systolic blood pressure and diastolic blood pressure at the beginning and end of the intervention or the differences of systolic blood pressure and diastolic blood pressure between the beginning and end of the intervention were reported; (v) the dose of grape polyphenols was provided.

### Data extraction and quality assessment

According to the predefined inclusion criteria, two reviewers (Shao-Hua Li and Peng Zhao) independently completed the search, data extraction, and quality assessment. Any discrepancies between the two reviewers were resolved through discussion until a consensus was reached. The extracted data included the study characteristics (authors, publication year, sample size, study design), population information (mean age, body mass index, healthy status), the dose of grape polyphenols supplementation, the duration of the intervention, and the systolic and diastolic blood pressure at the start and end of the intervention. If alcoholized and de-alcoholized red wine were both used as the supplementation of grape polyphenols, only the de-alcoholized data were extracted because the alcohol may also affect the blood pressure [22,23]. Meanwhile, if trial conducted two different doses of grape polyphenols (low-dose and high-dose) as the intervention, both the low-dose and high-dose data were extracted and separated into two independent trials (low-dose trial and high-dose trial).

The quality of the studies was judged by the following criteria: 1) randomization; 2) concealment of treatment allocation; 3) participant masking; 4) researcher masking; 5) reporting of withdrawals; 6) generation of random numbers; and 7) reporting of industry funding. Trials scored one point for each area addressed in the study design (randomization, blinding, concealment of allocation, reporting of withdrawals, and generation of random numbers), with a possible score of between 0 and 5 [24] (highest level of quality).

### Statistical analysis

The primary outcome was the overall changes of systolic and diastolic blood pressure between the beginning and the end of the intervention. If the changes in systolic and diastolic blood pressure were not reported in the study, we calculated them according to the Cochrane Handbook for Systematic Review and Follman D’s theory for overview of clinical trials with continuous variables [25]. We assumed equal variance among trials and between intervention and controls. Weighted mean differences (WMD) and 95% confidence intervals (CIs) were calculated for net changes in systolic and diastolic blood pressure by using fixed-effect models [26]. Statistic heterogeneity of treatment effects between studies was formally tested with Cochran’s test (*P* < 0.1). The *I* ^2^ statistic was also examined, and we considered an *I* ^2^ value >50% to indicate significant heterogeneity between the trials [27]. Furthermore, subgroup analyses were also performed to identify the possible sources of heterogeneity by comparing summary results obtained from subsets of studies grouped by age, body mass index, duration of intervention, health status of the subjects and the dose of grape polyphenols. Publication bias was assessed with the Egger regression test and Begg’s funnel plots [28]. Meta-analysis and statistical analyses were performed with Stata software (version 10.0; Stata Corporation, College Station, TX, USA) and REVMAN software (version 5.0; Cochrane Collaboration, Oxford, UK).

## Results

### Search results

**Fig 1:**
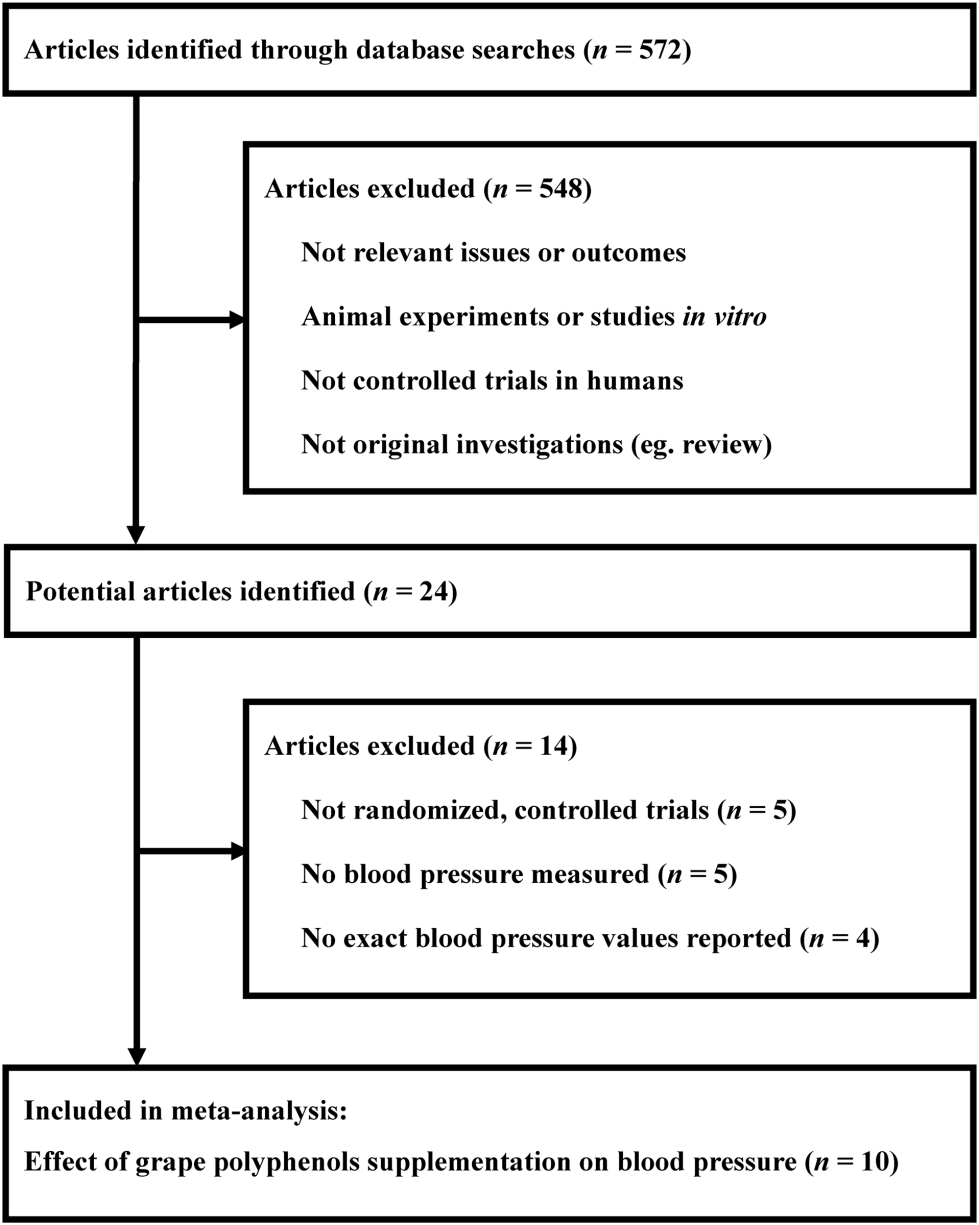
Identification process for eligible studies. Articles were initially identified in a combined search from PubMed, EMBASE, and Cochrane Library databases.

The flow chart of identification process is presented in Fig 1. A total of 572 articles were initially identified in a combined search of the PubMed, EMBASE, and Cochrane Library databases, of which 548 were excluded because they were studied in animals or *in vitro*, or because the objectives were not related to the present meta-analysis. The remaining 24 potentially relevant articles were examined for full text evaluation [10–19,22,29–41]. Of the 24 studies, 10 eligible randomized controlled trials were included in the present meta-analysis [10–19]. The other 14 articles were excluded for the following reasons: five studies were not randomized controlled design [29,31,32,35,36]; blood pressure was not measured in 4 trials [33,34,37,39]; the exact values of systolic and diastolic blood pressure at the start and end of the intervention or the change of systolic and diastolic blood pressure between the start and end of the intervention were not reported or calculated in five studies [22, 30, 38, 40, 41], but Hodgson *et al* [40] actually reported data in another article [15] which has been included in our present meta-analysis.

### Study characteristics

The characteristics of the 10 studies are presented in Table 1. All the 10 trials were randomized controlled, and 8 were double-blind design [11–15,17–19]. Of the 10 trials, 5 studies included healthy adults [10,12,16,18,19], and others enrolled hypertensive patients [15], subjects with high vascular risk [13] or metabolic syndrome [11,14], or patients with coronary disease [17]. The sample size of the 10 trials ranged from 9 to 70. The average age of the subjects ranged from 31.4 to 63.7 years old, and the body mass index varied from 23.2 kg/m2 to 36 kg/m2. Doses of grape polyphenols in these trials ranged from 150 mg/d to 1400 mg/d. The duration of treatment ranged from 2 weeks to 16 weeks.

**Table 1:** Characteristics of the included trials investigating the effects of grape polyphenols on blood pressure. R, randomized; SB, single-blind; DB, double-blind; PC, placebo-controlled; Con, controlled; CO, crossover; BMI, body mass index.

									Systolic blood pressure *		Diastolic blood pressure *	
Author	Year	Study Design	Health status of subjects	Number of subjects	Mean age (year)	BMI (kg/m^2^)	Duration	Dose of grape polyphenols	Grape polyphenols Baseline/Final(mmHg)	Control Baseline/Final(mmHg)	Grape polyphenols Baseline/Final (mmHg)	Control Baseline/Final (mmHg)
Clifton *et al*	2004	R DB Con CO	High vascular risk	Grape: 35 Control: 35	58	28.4	4 weeks	1000 mg/day	127±15 / 124±14	127±15 / 124±13	74±9 / 73±9	74±9 / 73±8
Ward *et al*	2005	R DB PC	Hypertensive patients	Grape: 16 Control: 18	62.5	28.5	6 weeks	1000 mg/day	133.6±11.6 / Not reported	134.1±8.2 / Not reported	79.8±9.8 / Not reported	78.2±7.1 / Not reported
Sano *et al* 200mg	2007	R SB PC	Healthy subjects	Grape: 18 Control: 18	52	24.2	12 weeks	200 mg/day	126.4±12.60 / 129.2±13.66	122.7±18.63 / 127.8±20.53	77.9±7.04 / 79.6±8.49	77.1±11.03 / 81.1±11.88
Sano *et al* 400mg	2007	R SB PC	Healthy subjects	Grape: 18 Control: 17	52	24.2	12 weeks	400 mg/day	126.2±16.41 / 127.7±12.37	122.7±18.63 / 127.8±20.53	78.0±10.14 / 79.5±8.16	77.1±11.03 / 81.1±11.88
Jimenez *et al*	2008	R Con	Non-smokers	Grape: 34 Control: 9	35.3	25.4	16 weeks	1400 mg/day	126.5±22.1/ 118±19.6	121.5±14.0 / 113.7±9.4	78.2±11.7 / 71.4±14.4	74.4±12.1 / 71.3±8.7
Sivaprakasapillai *et al* 150mg	2009	R DB PC	Metabolic syndrome	Grape: 9 Control: 9	46	36	4 weeks	150 mg/day	134±15 / 123±12	123±12 / 121±12	83±9 / 77±6	74±12 / 70±12
Sivaprakasapillai *et al* 300mg	2009	R DB PC	Metabolic syndrome	Grape: 9 Control: 9	46	36	4 weeks	300 mg/day	127±12 / 116±9	123±12 / 121±12	78±9 / 71±9	74±12 / 70±12
Mellen *et al*	2010	R DB PC CO	Coronary disease	Grape: 50 Control: 50	52.1	28.2	4 weeks	1300 mg/day	122.4±11.31 / 125.2±14.14	124.6±12.73 / 123.2±14.14	72.8±8.48 / 73.2±9.19	75.3±8.48 / 72.8±7.78
van Mierlo *et al*	2010	R DB PC CO	Healthy males	Grape: 35 Control: 35	31.4	23.2	2 weeks	800 mg/day	123±10.5 / 118±10.4	123±10.5 / 118.6±7.1	72.6±9.2 / 70.2±8.46	72.6±9.2 / 70.8±5.92
Barona *et al*	2012	R DB PC CO	Metabolic syndrome	Grape: 24 Control: 24	51.3	31.5	30 days	267 mg/day	131±10 / 122±11	131±10 / 128±10	87±9 / 83±8	87±9 / 84±9
Queipo-Ortuno *et al* De-alcoholized	2012	R DB Con CO	Healthy males	Grape: 10 Control: 10	48	27.6	20 days	733 mg/day	145.4±23.9 / 135.1±24.6	145.4±23.9 / 142.7±22.3	97.4±15.2 / 91.0±12.9	97.4±15.2 / 98.4±14.3
Ras *et al*	2013	R DB PC	Healthy subjects	Grape: 34 Control: 35	63.7	25.5	8 weeks	300 mg/day	135.8±11.08 / 130.3±9.91	135.7±10.06 / 132.5±10.06	81.9±8.75 / 79.1±7.58	81.1±7.10 / 80.0±6.51

* Values are the Mean ± SDs reported or calculated.

Two studies [11,16] used two different doses of grape polyphenols (low-dose and high-dose) as the interventions, so the low-dose and high-dose groups were separated into two independent trials in the present meta-analysis. Queipo-Ortuno's trial [12] designed an alcoholized and a de-alcoholized group to investigate the effect of grape polyphenols on blood pressure, and we only extracted the de-alcoholized data to our present meta-analysis because the alcohol could likely affect blood pressure [22,23]. The baseline and final values of systolic and diastolic blood pressure for the included trials are shown in Table 1.

The quality score of the 10 studies ranged from 1 to 4. Eight were randomized, double-blinded controlled studies [11–15,17–19], and one was single-blinded trial [16]. Six of the 10 studies reported the details of withdrawals [12,13,15,16,18,19], whereas the other four studies did not address this issue [10,11,14,17].

### The effect of grape polyphenols on blood pressure

**Fig 2:**
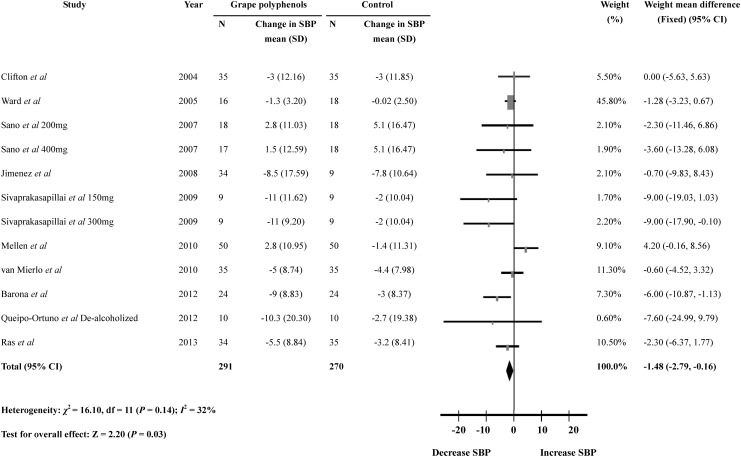
Meta-analysis of the effect of grape polyphenols on systolic blood pressure compared with controls. The sizes of the data markers indicate the weight of each study in the analysis.

Of the 10 trials, five showed a significant reduction in systolic blood pressure [10–14], other trials reported no significant difference in systolic blood pressure after grape polyphenols intervention [15–19]. The data of systolic blood pressure were extracted and pooled from the included studies, and the present meta-analysis showed that a significant reduction in systolic blood pressure by 1.48mmHg in the grape polyphenols-supplemented subjects than in control subjects (12 comparisons; WMD: -1.48mmHg; 95% CI: -2.79 to -0.16mmHg; *P* = 0.03) (Fig 2). No significant heterogeneity could be detected in the meta-analysis of systolic blood pressure (heterogeneity *I* ^2^ = 32%, *P* = 0.14). Subgroup analyses were performed to identify the possible sources of heterogeneity, and the results showed that systolic blood pressure was significantly reduced in low-dose of grape polyphenols (< 733 mg/day) when compared to the high-dose groups (*P* = 0.009). Meanwhile, the systolic blood pressure in metabolic syndrome patients (WMD:-7.05 mmHg, 95 CI: -10.97 to -3.12 mmHg) was also obviously decreased after intake of grape polyphenols when compared with other two subgroups (*P* = 0.03). No significant differences could be detected in other subgroup analyses (age, body mass index and duration). (S3 Table)

**Fig 3:**
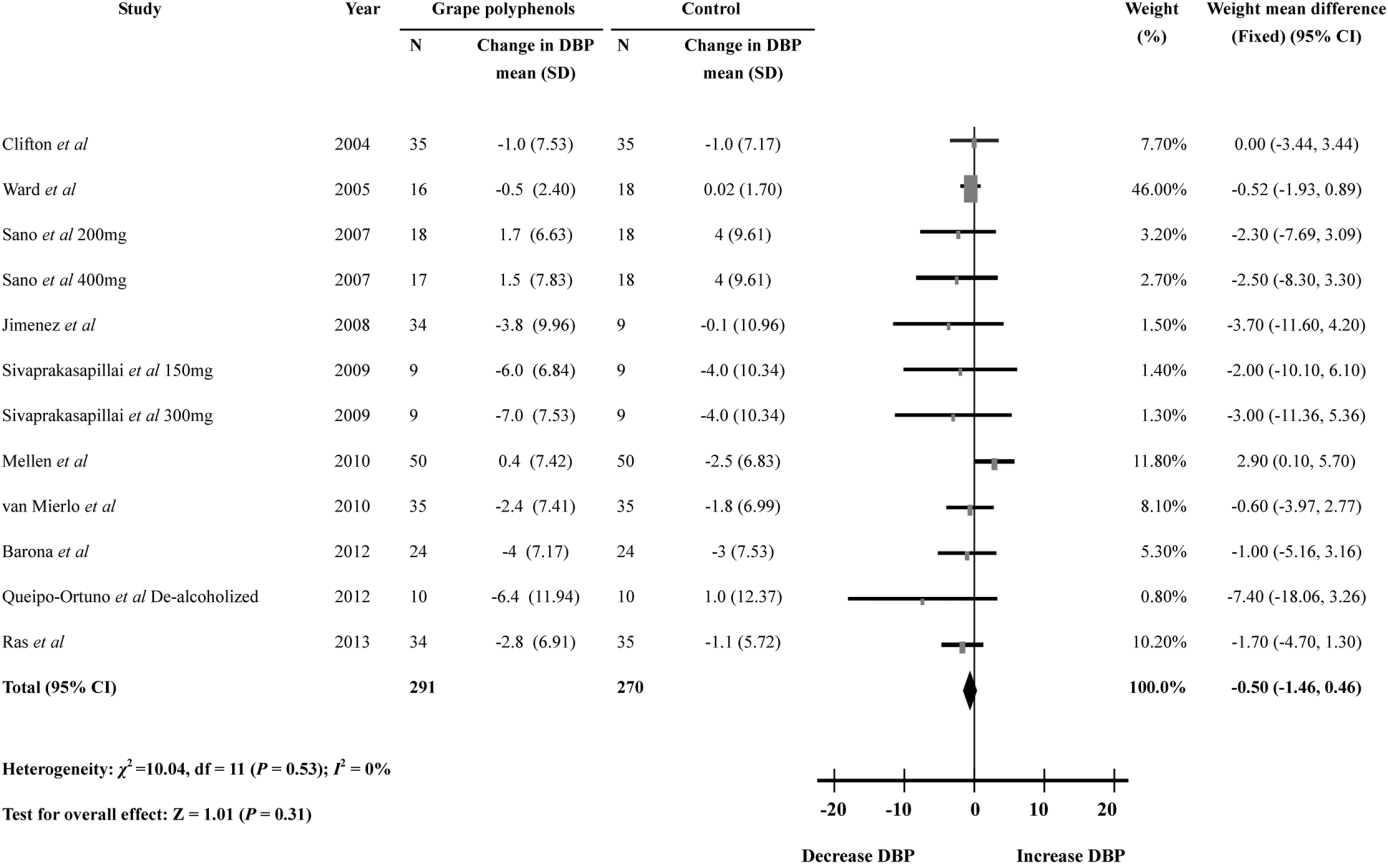
Meta-analysis of the effect of grape polyphenols on diastolic blood pressure compared with controls. The sizes of the data markers indicate the weight of each study in the analysis.

When estimating the diastolic blood pressure, three trials reported a significant reduction in diastolic blood pressure [10–12], and other seven trials reported no significant change of diastolic blood pressure after grape polyphenols supplementation [13–19]. The present meta-analysis was conducted and the result indicated that no significant reduction in diastolic blood pressure was observed between the grape polyphenols-supplemented subjects and controls (12 comparisons; WMD: -0.50mmHg; 95% CI: -1.46 to 0.46mmHg; *P* = 0.31) (Fig 3). Similarly, no heterogeneity could be found in the meta-analysis of diastolic blood pressure (heterogeneity *I* ^2^ = 0%, *P* = 0.53). Subgroup analyses were also performed but no significant differences could be detected in the analyses based on age, body mass index, duration, health status of the subjects or the dose of grape polyphenols. (S3 Table)

### Sensitivity analysis

Sensitivity analysis showed that the overall estimates of the changes in blood pressure were not altered after excluding the low-quality studies [10,16]. Exclusion of the low-quality studies [10,16] led to a small attenuation of the changes in blood pressure (systolic blood pressure: -1.43 [-2.79, -0.07], *P* = 0.04; diastolic blood pressure: -0.32 [-1.32, 0.67], *P* = 0.52), but the grape polyphenols intake still resulted in a significant reduction of systolic blood pressure.

### Publication bias

The publication bias of trials was assessed by the statistical analyses of the Egger test and funnel plots [28]. No publication bias was found in the meta-analysis of systolic blood pressure (Egger test, *P* = 0.234) and diastolic blood pressure (Egger test, *P* = 0.119).

## Discussion

The present meta-analysis showed that daily intake of grape polyphenols could significantly reduce systolic blood pressure by 1.48 mmHg when compared with control subjects. No significant heterogeneity publication bias could be detected in the meta-analysis of systolic blood pressure. Subgroup analysis indicated that larger reduction was identified in the intake of low-dose of grape polyphenols (< 733 mg/day, median level of the included studies) or patients with metabolic syndrome. In contrast, diastolic blood pressure was not significantly decreased in the grape polyphenols intervention group as compared with controls, and no significant heterogeneity or publication bias was found in the meta-analysis of diastolic blood pressure. Therefore, the present study supported the hypothesis that daily grape polyphenol intake might affect systolic blood pressure, but not diastolic blood pressure.

Significant increases of NO could be detected in human umbilical vein endothelial cells after being treated with grape polyphenol extract *in vitro* [42]. *In vivo* animal studies have further demonstrated that grape polyphenols induce an endothelium-dependent relaxation in rabbits, [8] and reduce arterial blood pressure in hypertensive rats [9], but the precise effects of grape polyphenols on blood pressure in humans has not been established. Feringa *et al* included 5 trials (until 2010) and conducted a meta-analysis investigating the effect of grape polyphenols on blood pressure [20], but one important trial published in 2008 [10] was not included in Feringa's meta-analysis, and the significant publication bias cloud be found in the meta-analysis of diastolic blood pressure. Moreover, some new trials investigating the effect of grape polyphenols on blood pressure have been published since 2010 [12,14,17,19], thus a new meta-analysis is needed to further explore the precise effect of grape polyphenols on systolic and diastolic blood pressure in humans. The present meta-analysis revealed that grape polyphenols intake resulted in a significant reduction of systolic blood pressure, but had no effect on diastolic blood pressure. The mean decrease of 1.48mmHg for systolic blood pressure was modest when compared with antihypertensive medications, but previous study revealed that a 4-5mmHg reduction in systolic blood pressure might significantly reduce cardiovascular risk by 8%- 20% [2].

The precise mechanisms of grape polyphenols in reducing blood pressure are currently unknown. The majority of studies suggested that the stimulation and promotion of the release of NO, resulting in the vasorelaxation, might be the main cause of hypotensive effect of grape polyphenols. Peng *et al*. found that the arterial blood pressure in hypertensive rats was significantly decreased after the supplementation of grape seed extract for 4 weeks [43]. Barona *et al*. further demonstrated that there was a negative association between the reduction of systolic blood pressure and the increased production of NO in patients with metabolic syndrome [14]. Our present meta-analysis included 10 randomized controlled trials and conducted a new meta-analysis, and the results showed that grape polyphenols intake in the daily life could significantly reduce the systolic blood pressure, but not diastolic blood pressure, consistent with the conclusions of Barona's study [14] and previous Feringa's meta-analysis [20]. Furthermore, our subgroup analyses showed that low-dose of grape polyphenols supplementation might lead to a significant reduction of systolic blood pressure. Edirisinghe *et al*. demonstrated that 1.0μmol/L grape seed extracts could induce the most significant relaxation of rabbit aortic rings *in vitro*, but no further prominent change of rabbit aortic rings could be found at higher concentrations (10μmol/L or 100μmol/L) of grape seed extracts [8], which might partially explain the results of our subgroup analysis. Meanwhile, our subgroup analysis also indicated that the systolic blood pressure in patients with metabolic syndrome was obviously decreased after intake of grape polyphenols, which might be ascribed to the impaired endothelial function in metabolic syndrome. Our previous meta-analysis [44] and other studies [38, 45] showed that intake of grape polyphenols could significantly improve the endothelial function, especially subjects with high cardiovascular risk factors. However, the exact mechanisms of grape polyphenols on blood pressure are still unclear and need to be further explored in the future.

Despite the interesting results of the present meta-analysis, several potential limitations should be addressed. First, the sample sizes in the included trials, which varied from 10 to 50 subjects, were still small; therefore, larger and better designed trials are needed to verify our conclusions in the future. Secondly, hypertensive patients enrolled in the present meta-analysis was limited; only one trial [15] included hypertensive subjects and other two trials enrolled patients with metabolic syndrome [11,14]. Whether the effect of grape polyphenols on blood pressure might be more significant in hypertensive subjects, especially in patients with severe hypertension, are needed to be confirmed in the future. Thirdly, lifestyle modifications during the intervention of grape polyphenols were not reported in majority of the included studies. The effect of grape polyphenols on blood pressure might be influenced by different lifestyle modifications and diets. Fourthly, although our subgroup analyses showed the low-dose of grape polyphenols supplementation might lead to a more significant reduction of systolic blood pressure, the doses of included trials varied from 150mg/day to 1400mg/day; therefore, more trials using other doses of grape polyphenols supplementation are needed to verify our present results. Fifth, although the mean reduction of 1.48 mm Hg for systolic blood pressure was statistically significant, the biological action generated from the effect of grape polyphenols has been not well clarified, might be less important than antihypertensive drugs, because some antihypertensive drugs have a well-established biological action on target organ damage irrespective of the antihypertensive effect. Therefore, the biological action of grape polyphenols should be well clarified in the future.

Conclusively, our present meta-analysis indicates that daily intake of grape polyphenols can significantly reduce systolic blood pressure in humans, although the reduction is modest when compared with antihypertensive medications. Larger, better designed studies that also include hypertensive subjects are required to verify our present results.

## Supporting Information

S1 Table The protocol of the present meta-analysis. This is the detailed protocol of the present meta-analysis.
(DOC)

S2 Table The PRISMA 2009 checklist of the present meta-analysis. This is the detailed checklist of the present meta-analysis according to the PRISMA guideline.
(DOC)

S3 Table Subgroup analyses for the effect of grape polyphenols on blood pressure. The subgroup analyses were performed and showed as supporting information.
(DOC)
