# Supplementary material for: Effect of Grape Polyphenols on Blood Pressure: A Meta-Analysis of Randomized Controlled Trials
Source: PLoS One. 2015 Sep 16;10(9):e0137665. doi: 10.1371/journal.pone.0137665 (PMC4572713; doi:10.1371/journal.pone.0137665)
Supplement: S1 Table — This is the detailed protocol of the present meta-analysis. (DOC) [file pone.0137665.s001.doc]

**S1 Table The protocol of the present meta-analysis**

***Review question(s)***

How do grape polyphenols intake in daily life impact on systolic and diastolic blood pressure in humans?

***Searches***

We systematically searched PubMed, EMBASE, and the Cochrane Library for published reports by using the query "(grape) OR (polyphenol) " paired with "(blood pressure) OR (hypertension)". In addition, a manual search of references from reports of clinical trials or review articles was performed to identify relevant trials. Studies were selected for analysis if they met the following criteria:

(i) The article was published in English;

(ii) Study was randomized and controlled trial in humans;

(iii) The subjects in the trial were exposed to the intervention for a minimum of 2 weeks;

(iv) Means of systolic blood pressure and diastolic blood pressure at the beginning and end of the intervention or the differences of systolic blood pressure and diastolic blood pressure between the beginning and end of the intervention have been reported;

(v) The dose of grape polyphenols was provided.

***Types of study to be included***

We will identify all published, randomized and controlled trials of grape polyphenols and perform a meta-analysis to evaluate the effect of grape polyphenols on systolic and diastolic blood pressure..

***Condition or domain being studied***

Blood pressure is a pivotal parameter for cardiovascular system and hypertension has become an important risk factor for cardiovascular diseases. Previous study has been revealed that a 4-5 mmHg reduction in systolic blood pressure and a 2-3 mmHg reduction in diastolic blood pressure can significantly reduce the cardiovascular risk by 8% - 20%.

***Participants/ population***

Inclusion:

(1) The subjects should be exposed to the intervention (grape polyphenols or placebo) for a minimum of 2 weeks;

(2) The systolic and diastolic blood pressure should be measured at the beginning and end of the intervention in the subjects.

Exclusion:

(1) The subjects were exposed to the intervention (grape polyphenols or placebo) less than 2 weeks;

(2) The systolic and diastolic blood pressure were not measured in the subjects;

(3) Subjects were excluded if they had a familial or personal history of psychiatric disorders, epilepsy and sleep disorders.

***Intervention(s), exposure(s)***

Grapes polyphenols, such as flavans, anthocyanins, flavonols, and stilbenes (resveratrol), have been demonstrated to modulate LDL oxidation, oxidative stress, dyslipidemia, and inflammation. The grape polyphenols control interventions are performed in humans that were given food or drink containing grape polyphenols extract (intervention group) or similar food or drink without grape polyphenols extract (control group) for several weeks ( > 2 weeks). The systolic blood pressure and diastolic blood pressure at the beginning and end of the intervention were measured in the participants. Blood pressure was measured on the arm at the heart level after at least 5 min of resting in sitting position and using an blood pressure monitor. Two recordings, separated by at least 1 min were made and the mean value was used. If there was more than a 5-mm Hg difference between the first and second readings, additional readings were obtained and then the mean value of these multiple readings was used.

***Comparator(s)/ control***

A non-exposed control group

Control group should be given similar drinks or food to the intervention group but without grape polyphenol extract.

***Outcomes***

Primary outcomes

The primary outcome was the overall changes of systolic and diastolic blood pressure between the beginning and end of the intervention.

***Data extraction, (selection and coding)***

According to the predefined inclusion criteria, two reviewers independently completed the search, data extraction, and quality assessment. Any discrepancies between the two reviewers were resolved through discussion until a consensus was reached. The extracted data included the study characteristics (authors, publication year, sample size, study design), population information (mean age, body mass index, healthy status), the dose of grape polyphenols supplementation, the duration of the intervention, and the systolic and diastolic blood pressure at the start and end of the intervention.

***Risk of bias (quality) assessment***

The risk of bias in included studies will be assessed by two review authors considering the following characteristics:

Treatment allocation concealment: was the allocated treatment adequately concealed from study participants and clinicians and other healthcare or research staff at the enrolment stage?

Blinding: were the personnel assessing outcomes and analyzing data sufficiently blinded to the intervention allocation throughout the trial?

Completeness of outcome data: were participant exclusions, attrition and incomplete outcome data adequately addressed in the published report?

Selective outcome reporting: is there evidence of selective outcome reporting and might this have affected the study results?

Publication bias: Publication bias will be assessed with the Egger regression test and funnel plots. Disagreements between the review authors over the risk of bias in particular studies will be resolved by discussion, with involvement of a third review author where necessary.

***Strategy for data synthesis***

We will provide a narrative synthesis of the findings from the included studies, structured around the type of intervention (controlled trial), target population characteristics, type of outcome (the changes of systolic and diastolic blood pressure due to the intervention of grape polyphenols supplementation) and intervention content. We will provide summaries of intervention effects for each study by calculating mean differences.

We anticipate that meta-analysis can be performed because of the outcome measured across the small number of existing trials. However, where studies have used the same type of intervention and comparator, with the same outcome measure, we will pool the results using random-effects or fixed-effects model for meta-analyses, with mean differences for blood pressure. Weighted mean differences and 95% confidence intervals (CIs) will be calculated for net changes in blood pressure. Statistical heterogeneity of treatment effects between studies will be formally tested with Cochrane’s test (*P* < 0. 1). The *I*^2^ statistic will also be examined, and we will consider an *I*^2^ value > 50% to indicate significant heterogeneity between the trials. Potential heterogeneity in estimates of treatment effect will be explored by univariate meta-regression or subgroup analyses. Publication bias will also be assessed with the Egger regression test and funnel plots.

***Analysis of subgroups or subsets***

If the necessary data are available, subgroup analyses will be done for people with healthy status, the dose of grape polyphenols supplementation, or the duration of the intervention.
