# Supplementary material for: Effect of Grape Polyphenols on Blood Pressure: A Meta-Analysis of Randomized Controlled Trials
Source: PLoS One. 2015 Sep 16;10(9):e0137665. doi: 10.1371/journal.pone.0137665 (PMC4572713; doi:10.1371/journal.pone.0137665)
Supplement: S3 Table — The subgroup analyses were performed and showed as supporting information. (DOC) [file pone.0137665.s003.doc]

**S3 Table Subgroup analyses for the effect of grape polyphenols on blood pressure**

|  | Systolic blood pressure | | |  | Diastolic blood pressure | | |
| --- | --- | --- | --- | --- | --- | --- | --- |
|  | Intervention group | Effect (95% CI) | *P* |  | Intervention group | Effect (95% CI) | *P* |
| Age |  |  |  |  |  |  |  |
| < 52 years, low median | 6 | -3.64 (-6.27, -1.02) | 0.06 |  | 6 | -1.54 (-3.78, 0.69) | 0.31 |
| ≥ 52 years, high median | 6 | -0.75 (-2.27, 0.78) |  |  | 6 | -0.26 (-1.32, 0.80) |  |
| Body mass index |  |  |  |  |  |  |  |
| < 28.2 kg/m^2^, low median | 6 | -1.70 (-4.17, 0.78) | 0.84 |  | 6 | -1.80 (-3.67, 0.06) | 0.11 |
| ≥ 28.2 kg/m^2^, high median | 6 | -1.39 (-2.95, 0.17) |  |  | 6 | -0.03 (-1.14, 1.09) |  |
| Duration |  |  |  |  |  |  |  |
| ≤ 4 weeks, low median | 6 | -0.27 (-2.66, 2.12) | 0.23 |  | 6 | 0.53 (-1.19, 2.25) | 0.16 |
| > 4 weeks, high median | 6 | -2.01 (-3.58, -0.43) |  |  | 6 | -0.96 (-2.12, 0.19) |  |
| Dose of grape polyphenols |  |  |  |  |  |  |  |
| < 733 mg, low median | 6 | -4.48 (-7.08, -1.87) | 0.009 |  | 6 | -1.80 (-3.75, 0.15) | 0.13 |
| ≥ 733 mg, high median | 6 | -0.44 (-1.97, 1.09) |  |  | 6 | -0.08 (-1.18, 1.02) |  |
| Health status |  |  |  |  |  |  |  |
| Healthy | 6 | -1.70 (-4.17, 0.78) | 0.03 |  | 6 | -1.80 (-3.67, 0.06) | 0.54 |
| Hypertensive* | 1 | -1.28 (-3.23, 0.67) |  |  | 1 | -0.52 (-1.93, 0.89) |  |
| Metabolic syndrome | 3 | -7.05 (-10.97, -3.12) |  |  | 3 | -1.50 (-4.89, 1.88) |  |

* Not pure hypertensive subjects enrolled in Clifton’s and Mellen’s trials, thus the two trials were not included in the subgroup analysis.
